# The role of neurotrophins in psychopathology and cardiovascular diseases: psychosomatic connections

**DOI:** 10.1007/s00702-019-01973-6

**Published:** 2019-02-14

**Authors:** Andrea László, Lilla Lénárt, Lilla Illésy, Andrea Fekete, János Nemcsik

**Affiliations:** 1 Department of Family Medicine, Semmelweis University, Budapest, Hungary; 2 First German Hospital for Traditional Chinese Medicine, Bad Kötzting, Germany; 3 MTA-SE “Lendület” Diabetes Research Group, Budapest, Hungary; 4 MTA-SE Pediatrics and Nephrology Research Group, Budapest, Hungary; 5 Health Service of Zugló (ZESZ), Budapest, Hungary

**Keywords:** Neurotrophic factors, Mood disorders, Cardiovascular diseases, Psychosomatic connections

## Abstract

Cardiovascular (CV) diseases and mood disorders are common public health problems worldwide. Their connections are widely studied, and the role of neurotrophins (NTs) is already supposed in both conditions. However, data in the literature of clinical aspects are sometimes controversial and no reviews are available describing possible associations between CV risk and mood disorders based on NTs. The mostly studied NT is brain-derived neurotrophic factor (BDNF). Decreased level of BDNF is observed in depression and its connection to hypertension has also been demonstrated with affecting the arterial baroreceptors, renin–angiotensin system and endothelial nitric oxide synthase. BDNF was also found to be the predictor of CV outcome in different patient populations. Other types of human NT-s, such as nerve growth factor, neurotrophin 3 and neurotrophin 4 also seem to have both psychopathological and CV connections. Our aim was to overview the present knowledge in this area, demonstrating a new aspect of the associations between mood disorders and CV diseases through the mediation of NTs. These findings might enlighten new psychosomatic connections and suggest new therapeutic targets that are beneficial both in respect of mood disorders and CV pathology.

## Introduction

Neurortophic factors or neurotrophins (NTs) consist a family of trophic factors of secreted proteins that promote growth, survival and differentiation of neurons both in the central and peripheral nervous system (Chao et al. 2006). The members of the NT family are structurally similar proteins (Keefe et al. 2017), and in mammals comprise the following four types: brain-derived neurotrophic factor (BDNF), nerve growth factor (NGF), neurotrophin 3 (NT-3), and neurotrophin 4 (NT-4) (Skaper 2008). The biological effects of mature NTs are mediated through the activation of one or more of the three tyrosine kinase receptors: tropomyosin receptor kinase A, -B, -C (TrkA, TrkB, TrkC). Nevertheless, NTs are synthesized from proneurotrophins, which are proteolytically cleaved to mature NTs. Proneurotrophins preferentially activate p75 neurotrophin receptor (p75Ntr) (Skaper 2008) and mainly induce apoptotic processes. The proneurotrophin cleavage seems to be an important factor, as the two classes of receptors (Trks and p75Ntr) interestingly act antagonistically on many physiological functions (Tsai 2017).

Both transcriptional and posttranslational mechanisms are involved in upstream regulation of neurotrophins. Neurotrophins’ gene structure is very complex, contains several coding and non-coding exons with multiple promoters and various spliced variants have also been reported. Human BDNF, for example, has a very complex gene structure, including 11 non-coding exons that are spliced independently to form a coding exon, thus more than 15 mRNA transcripts can be produced (Pruunsild et al. 2007). There are several cis and trans-acting transcriptional elements as well, which regulate the promoters of neurotrophins, contributing to various productions of neurotrophins. In neurons, cAMP response element binding protein (CREB) is one of the major transcription factors and an important regulator of neurotrophins’ gene expression.

Other studies demonstrated that various neurotransmitters, hormones and other neurotrophins also contribute to the transcriptional regulation of neurotrophins, but the molecular components involved in this regulation have not been clarified yet (Lindholm et al. 1994). Glutamate and acetylcholine up-regulate NGF and BDNF mRNA expression, while GABA down-regulates the levels of NGF and BDNF (Zafra et al. 1990, 1991). The regulation of NT-3 production is independent of cholinergic neuronal activity (da Penha Berzaghi et al. 1993).

Posttranslational modifications are also important processes regulating the productions of neurotrophins. Recently, a direct link between sigma-1 receptor (S1R) and BDNF has been discovered. Some studies described that S1R agonists (endogen like dehydroepiandrosterone or exogenous like SSRI fluvoxamine) increase BDNF expression and activate its downstream signaling. Others demonstrated that S1R acting as a chaperone protein enhances secretion of mature BDNF (Fujimoto et al. 2012). Our group also showed that S1R agonism by the fluvoxamine increases BDNF secretion in the rat hippocampus (Lenart et al. 2016).

Cardiovascular (CV) diseases are the leading cause of morbidity and mortality in most industrialized countries worldwide, despite highly effective preventive treatments. As CV diseases exert an excessive public health burden, exploring new pathophysiological pathways with the hope of new preventive and therapeutic potential can have an outmost importance. One of these new possibilities could be the involvement of NTs, which exert diverse effects on the developing and mature CV system. Their expression continues throughout life, influencing hypertension, atherosclerosis, diabetes and myocardial ischemia (Emanueli et al. 2014).

Mood disorders are also common public health problems in the Western world and their strong connection with CV diseases is broadly recognized (Penninx et al. 2001). Lower NT concentrations, such as serum BDNF and NGF have been shown to correlate negatively with many affective disorders including bipolar disorder (Barbosa et al. 2014; Lin et al. 2014), major depressive disorder (Brunoni et al. 2008), mania (Tramontina et al. 2009) and obsessive compulsive disorder (Maina et al. 2010). Although NTs themselves do not control mood directly, they are fundamental in the activity-dependent modulation of networks and changes in plasticity can affect mood as well (Castren et al. 2007).

As NTs can cross the blood–brain barrier, they potentially can influence CV pathology and psychopathology at the same time. BDNF is not only able to cross the blood–brain barrier (Pan et al. 1998), but in the periphery it is stored in platelets as well (Karege et al. 2002). Interestingly, while BDNF and NT-3 (as well as NT-4) are both structurally and functionally related to NGF (Maness et al. 1994; Sariola et al. 1994), the permeability coefficient-surface area of NGF is much lower compared with other NTs (Poduslo and Curran 1996).

Our aims are to overview the changes of NTs in psychiatric conditions and in CV diseases to demonstrate a new area of psychosomatic connections. We also aim to review the present knowledge about the therapeutic possibilities to restore the level of NTs and their consequences for different outcomes.

### Brain-derived neurotrophic factor (BDNF)

BDNF is the most frequently studied NT. It is initially synthesized in the endoplasmic reticulum as its precursor protein, preproBDNF. After the cleavage of the signal peptide this form becomes proBDNF, and proBDNF is converted by extracellular proteases to mature BDNF (Ethell and Ethell 2007; Yoshida et al. 2012).

Mature BDNF binds with higher-affinity Trk family receptors (Villanueva 2013)—especially TrkB—increasing cell survival and differentiation, dendritic spine complexity, long-term potentiation (Lu et al. 2008; Messaoudi et al. 2002), synaptic plasticity (Kang and Schuman 1996), and the resculpting of neuronal networks (Lee et al. 2001). ProBDNF is also biologically active, it mediates its actions through binding to low-affinity p75Ntr, having antagonistic effect compared to matured BDNF: reducing spine complexity and density (Zagrebelsky et al. 2005) and promoting neuronal cell death (Teng et al. 2005).

#### BDNF in mood disorders

Numerous clinical studies confirm the involvement of BDNF in the pathophysiology of depression (Lee and Kim 2010). Reductions in serum and plasma mature BDNF have been demonstrated in patients suffering from depression (Lee et al. 2007; Yoshida et al. 2012) and in those who committed suicide (Birkenhager et al. 2012; Kim et al. 2007b). Significantly lower levels of serum BDNF were found in antidepressant-free patients with major depressive disorder compared with healthy controls (Shimizu et al. 2003), which findings were confirmed by a large cohort study (Molendijk et al. 2011) and by three meta-analyses as well (Bocchio-Chiavetto et al. 2010; Brunoni et al. 2008; Sen et al. 2008).

The regulatory background of BDNF in psychopathology is not totally clarified yet, but epigenetic mechanisms (chromatin remodeling, DNA methylation) have been implicated. Both in schizophrenia and in depression at the promoter I of BDNF DNA methylation at a specific CpG site is increased resulting in reduced BDNF gene transcription (Fuchikami et al. 2011; Ikegame et al. 2013).

Single nucleotide polymorphism in BDNF gene is a plausible candidate to be associated with the development of psychopathology. The Val*66*Met polymorphism is characterized by an amino acid substitution of valine (Val) to methionine (Met) at amino acid residue 66. The presence of Met allele causes a failure in intracellular trafficking and packaging of proBDNF, which results in a 25% reduction of the activity-dependent secretion of mature BDNF (Egan et al. 2003). However, the role of Val*66*Met polymorphism in psychiatric disorders is well studied but the results are inconsistent. Some studies reported that Met/Met patients have more severe symptoms of depression (Czira et al. 2012), whereas others showed that Val/Val genotype is associated with increased depressive symptoms (Jiang et al. 2013) or higher scores on the cognitive–affective factor of the Beck Depression Inventory-II test (Duncan et al. 2009).

#### BDNF and CV pathology

BDNF plays an important role during development of the CV system: it activates TrkB receptor leading to the survival of endothelial cells and the formation of the cardiac vasculature (Emanueli et al. 2014). Embryonic BDNF deficiency impairs the development of intramyocardial vessels and can also lead to cardiac hypercontractility (Donovan et al. 2000). BDNF functions as an angiogenic regulator, promoting angiogenesis (Kermani et al. 2005). It is expressed in a greater amount in the peripheral vessels, where it could influence vasoreactivity (Prigent-Tessier et al. 2013). BDNF is able to enhance vascular flow and can regulate revascularization of ischemic tissues (Kermani et al. 2005). Furthermore, it improves left-ventricular function in ischemic myocardium (Liu et al. 2006).

In CVD, the role of Val*66*Met polymorphism is still unclear, but interestingly in a follow-up clinical study patients carrying Met allele were associated with a reduced risk of clinical CVD events and lower severity of coronary artery disease than Val/Val genotype (Jiang et al. 2017). However, further investigations are needed to clarify the genetic aspect of the link between BDNF and CVD.

How can BDNF influence blood pressure, potentially playing an important role in the development of hypertension? Axonal guidance is among the top pathways explaining the association between mood disorders and cardio-metabolic-disease risk (Amare et al. 2017). Mutant axonal guidance genes—including BDNF—followed by abnormal axonal guidance and connectivity can cause disorders primarily in the brain and subsequently in peripheral organs (Sasaki et al. 2014). During embryonic development, BDNF is found to be a target-derived survival factor for a large subset of nodose ganglion neurons, such as arterial baroreceptors (Brady et al. 1999) and is also involved in the development of chemoafferent sensory neurons innervating the carotid body (Conover et al. 1995; Erickson et al. 1996). Furthermore, postnatally BDNF is expressed by the nodose ganglion neurons themselves (Schecterson and Bothwell 1992; Wetmore and Olson 1995) and can be also released from these neurons by activity (Balkowiec and Katz 2000). BDNF is expressed in arterial baroreceptors and their central terminals in medial nucleus tractus solitarius in vivo. BDNF release from cultured nodose ganglion neurons is increased by electrical stimulation with patterns that mimic the in vivo activity of baroreceptor afferents (Martin et al. 2009). Thus it seems that BDNF is involved not only in the development of baroreceptors, but also in their normal functioning in adulthood.

During normal conditions when blood pressure increases, the activated baroreflex reduces heart rate and blood pressure by a negative feedback loop. In addition, elevated blood pressure activates inhibitory GABAergic neurons in the hypothalamus, reducing the secretion of the blood pressure-elevating hormone vasopressin (Marosi and Mattson 2015). But different pathophysiological changes can influence the mechanism of the baroreflex loop. It is already shown that high dietary salt intake can affect blood pressure through NT-mediated changes of the central homeostatic circuit. Choe et al. proved in an animal study that chronic high salt intake is able to decrease the baroreceptor-mediated inhibition of vasopressin neurons through a BDNF-dependent activation of TrkB receptors and through the downregulation of potassium/chloride co-transporter 2 expression, which prevents inhibitory of GABAergic signaling (Choe et al. 2015). Furthermore, reduced BDNF level in mice results in elevated heart rate, and infusion of this NT into the cerebral ventricles can restore this effect (Wan et al. 2014). In the same study, Wan et al. showed that GABAergic responses are increased in brainstem cardiovagal neurons of BDNF+/− mice, suggesting that BDNF increases the activity of the parasympathetic neurons to reduce heart rate (Wan et al. 2014). In summary, BDNF is required for normal carotid body innervation, baroreceptor function and heart rate regulation and these effects can be blunted in pathological conditions, such as high salt intake, which can lead to the development of hypertension.

Another pathway explaining the association between BDNF, CV function and susceptibility to mental diseases as well is the renin–angiotensin system (RAS). Increased central RAS activation is an indicator of many CV diseases, such as hypertension and heart failure (Biancardi et al. 2014; Zucker et al. 2012). On the other hand, data are accumulating about the newly discovered effects of the RAS related to neuroprotection, cognition and cerebral vasodilation. Angiotensin (AT) 1–7 also affects non-CV functions in the brain, such as learning, memory, and neuroprotection (Farag et al. 2017). Clinical studies have shown that AT II receptor type 1 (AT1R) blockers—independent of blood pressure-lowering effect—improve cognitive function in elderly hypertensive patients (Fogari et al. 2003; Hajjar et al. 2005). The background mechanism of this phenomenon was investigated in animal studies. Goel et al. showed the evidence that chronic neuroinflammation and memory impairment in hypertension—associated with increased apoptotic cell death and with amyloid beta deposition—can be prevented with candesartan treatment, suggesting partly to be explained by an increase of BDNF/CREB (cAMP response element binding protein) expression (Goel et al. 2017). Furthermore, the connection between RAS and TrkB signaling is proven in vitro (Becker et al. 2015) and in vivo as well, as Becker et al. demonstrated the mediator role of BDNF-TrkB signaling on Ang II-induced mean blood pressure and renal sympathetic nerve activity elevation in male rats (Becker et al. 2017). Thus probably RAS blockers restore BDNF through TrkB signaling pathway.

Cumulating data suggest the connection between endothelial dysfunction and BDNF as well. In an animal study, the protecting effect of the AT1R blocker candesartan after stroke was mediated by endothelial nitric oxide (NO) synthase and it was positively associated with BDNF expression (Alhusban et al. 2017). BDNF is probably indirectly associated with the NO-system as BDNF is secreted by endothelial cells (Zoladz and Pilc 2010), it increases vascular endothelial growth factor (VEGF) expression, which induces angiogenesis (Chen et al. 2005a; Lin et al. 2014) and VEGF also enhances the NO production of endothelial cells (Youn et al. 2009). The connection with endothelial dysfunction is also supported by the observation, that circulating BDNF level inversely correlates with vascular cell adhesion molecule-1 (Lee et al. 2012), which is an accepted biomarker of endothelial dysfunction (Burger and Touyz 2012).

Numerous data are available about the association between BDNF and CV health. In general population, a significant positive correlation was observed between plasma BDNF and diastolic blood pressure, and sexual differences were demonstrated in relation with different serum lipids (Golden et al. 2010). As it was a cross-sectional study, it is unclear if these associations observed are casual or elevated plasma BDNF represents a compensatory response of the disrupted lipid metabolism and hypertension, but the elevation of serum BDNF in hypertension was confirmed by our study as well (Nemcsik et al. 2016). Interestingly, in contrast, decreased endothelial BDNF expression was found in hypertension (Prigent-Tessier et al. 2013), so the source of higher BDNF serum and plasma levels in hypertension is probably not the endothelial cells. Increased BDNF expression was found in atherosclerotic coronary arteries in humans (Ejiri et al. 2005), and decreased plasma BDNF level was observed in patients with metabolic syndrome (Chaldakov et al. 2004), acute coronary syndrome (Lorgis et al. 2010; Manni et al. 2005) and in type 2 diabetes mellitus (Krabbe et al. 2007).

About obesity we found contradictory results in the literature. Previous studies showed that serum BDNF is generally lower in overweight and obese subjects (Celik Guzel et al. 2014; Corripio et al. 2012; El-Gharbawy et al. 2006). In contrary, others showed elevated serum BDNF level in obesity (Roth et al. 2013). This phenomenon has been explained by the fact that BDNF is also regulated by leptin, therefore leptin resistance—as one of the obesity-associated factors—shall be considered in future studies.

Serum BDNF level was diminished in smokers, compared to non-smokers (Bhang et al. 2010; Kim et al. 2007a). Furthermore, BDNF levels in chronic smokers were increased after smoking cessation (30).

In patients with angina pectoris Jiang at al. found that plasma BDNF was inversely associated with triglyceride and low-density lipoprotein (LDL)-cholesterol, male sex and age, while it was correlated positively with high-density lipoprotein (HDL)-cholesterol. In this cohort, low plasma BDNF was an independent predictor of future coronary events and mortality (Jiang et al. 2011). The predictive role of BDNF for future CV events and mortality was confirmed by other studies as well. Higher seBDNF was found to be associated with decreased risk of CV morbidity and mortality (Kaess et al. 2015). On contrary, decreased serum BDNF was found to be associated with increased risk of incident stroke/TIA (Pikula et al. 2013). In summary, these results suggest that BDNF is compensatory elevated in CV pathology and the lack of this elevation bears propensity for poor outcome.

#### Direct psychosomatic connections of BDNF

Only a few studies are available evaluating the role of BDNF both in vascular and psychiatric conditions. Affective temperament types (depressive, cyclothymic, hyperthymic, irritable and anxious) are subclinical, trait-related manifestations and commonly the antecedents of minor and major mood disorders (Rihmer et al. 2010). We previously demonstrated that in chronic hypertensive patients with dominant irritable, anxious, cyclothymic and depressive affective temperaments, serum BDNF level was decreased compared with chronic hypertensive patients without dominant affective temperaments (Laszlo et al. 2015). In chronic hypertensive patients, hyperthymic temperament score was found to be associated positively with serum BDNF level (Nemcsik et al. 2016). As hyperthymic temperament score was found to be inversely associated with coronary atherosclerosis (Nemcsik et al. 2017), it can be proposed that BDNF is a mediator of the protective effect of hyperthymic temperament in CV conditions. But in hypertension the elevation of BDNF can also be an effect of medications as in a preliminary study after 3 months of antihypertensive therapy a tendency of BDNF increase was observed (Korosi et al. 2017). In this cohort, almost all patients did get the ACE-inhibitor perindopril, which, although only in the brain, but was demonstrated to influence BDNF (Ali et al. 2016).

#### Opportunities to restore BDNF level

Considering that BDNF is involved in CV physiology and through enhancing the neuroplasticity and neurogenesis, it increases the resistance of neurons to metabolic and excitotoxic stress (Marosi and Mattson 2014) a new therapeutic target of mood and CV disorders could be the restoration of BDNF level.

Lifestyle changes like physical activity, such as running and other types of aerobic exercise (Engesser-Cesar et al. 2007; Griffin et al. 2011) or calorie restriction (Marosi and Mattson 2014) could be cardioprotective through BDNF mediation.

Long-term treatment with various antidepressants can also normalize serum BDNF level (Duman and Monteggia 2006; Sen et al. 2008). In animal studies, antidepressants, including selective serotonin reuptake inhibitors, selective norepinephrine reuptake inhibitors, and monoamine oxidase inhibitors elevate BDNF mRNA level in hippocampus (Huang et al. 2008). In psychiatry practice, BDNF level improvement can be evoked not only through medication, but also through electroconvulsive therapy (Brunoni et al. 2014). In relation with the CV pharmacology, the AT1R blocker candesartan is proven to restore BDNF (Alhusban et al. 2017) and the ACE-inhibitor perindopril has beneficial effects as well (Ali et al. 2016), but interestingly in case of ramipril this feature seems to be missing (Krikov et al. 2008). As we previously mentioned, RAS blockers probably restore BDNF through TrkB signaling pathway.

In the future, a possibility of BDNF restoration could be the inhibition of its degradation. The mechanism of BDNF degradation is not well investigated, in the literature there are only few studies about this process. More than 25% of synthesized BDNF is depredated by lysosomes. Soluble sortilin is a main protein, which directs the trafficking of BDNF. Sortilin binds to sorting motif of BDNF and facilitates BDNF allocation to the late endosome; hereby sortilin rescues BDNF from lysosomal degradation. Until now no pharmacological option exists to inhibit the degradation of BDNF. Modifying sortilin either with increasing its level or its binding action would be options to increase total BDNF levels through its decreased targeting to the lysosome (Evans et al. 2011).

As there is no agent that would reduce BDNF degradation, direct receptor (TrkB) activation via ligands/agonists or mechanisms of increasing the BDNF level would be also appropriate therapeutic applications. Based on the listed psychopathological and CV effects of BDNF, such a medication can potentially be beneficial for both systems.

### Nerve growth factor (NGF)

NGF is another important member of the NT family, produced mainly in the cortex, hippocampus, hypothalamus, but—like BDNF—it is also found in peripheral nervous and immune systems (Wiener et al. 2015). The adrenal gland also appears to be an important biological target of NGF (Alleva et al. 1993; Aloe et al. 1986), thus it could represent an intermediate station in the communication between central nervous system structures and peripheral organs (Cirulli and Alleva 2009). The mature, active form of NGF descends from proteolytic cleavage of a precursor form proNGF, which also has important roles during development and in adult life, having both pro-apoptotic and neurotrophic properties (Fahnestock et al. 2004a, b). The biological action of NGF is mediated by two receptors: the high-affinity receptor TrkA, and the low-affinity transmembrane glycoprotein receptor p75, that regulates signaling through TrkA (Huang and Reichardt 2003). Binding of NGF to p75Ntr activates additional signaling pathways: in the absence or reduced expression of coexpressed TrkA receptors on NGF-target cell apoptosis is triggered by NGF (Ebendal 1992; Huang and Reichardt 2003). Therefore, the effect of NGF on target cells depends on the ratio of these two receptors co-distributed on the cell surface (Micera et al. 2007).

#### NGF in mood disorders

Like BDNF, NGF seems to be negatively associated with depression and suicide. Clinical studies showed reduced level of NGF in patients with major depressive disorder compared with healthy controls (Diniz et al. 2013; Xiong et al. 2011), and a study with suicide victims showed also decreased NGF in the hippocampus (Banerjee et al. 2013). Comparing patients with major depressive disorder to such with major depressive disorder and additional suicide risk, Wiener et al. found no differences in NGF levels, but differences were found when comparing them to healthy controls (Wiener et al. 2015). Similarly, Dwivedi et al. found differences of NGF levels in the prefrontal cortex and in the hippocampus only when comparing suicide victims with major depressive disorder and suicide victims with other psychiatric disorders to healthy controls, but not when comparing the two analyzed groups to each other (Dwivedi et al. 2005). On the contrary, in an elderly population, Ziegenhorn et al. did not confirm the influence of depression on serum NGF level (Ziegenhorn et al. 2007). Thus, it seems that NGF has an important role in the pathophysiology of mood disorders, but with aging this association can be attenuated.

#### NGF and CV pathology

Additionally to neural growth effects, NGF was the first NT found to be involved in the postnatal angiogenesis with the mediation of TrkA receptors (Emanueli et al. 2014)—although the mechanism of the angiogenic effects of NGF is not totally clarified yet. It is mediated either by a direct effect on vascular endothelial cells, or indirectly by influencing the action of other endogenous growth factors, for example, VEGF (Han et al. 2008; Nico et al. 2008). Asanome et al. showed in an animal study that the effects of NGF on vascular maturation are mediated through perivascular nerves (Asanome et al. 2014). Among other NTs, the expressions of NGF and TrkA are both dramatically upregulated by arterial balloon injury in rats and during neointima formation their increased levels are maintained (Emanueli et al. 2014).

It is known since the 90s that in spontaneously hypertensive rats the NGF secretion in the vessels is elevated (Spitsbergen et al. 1998; Tuttle et al. 1995; Zettler and Rush 1993). It is associated with sympathetic hyperinnervation (Head 1989; Spitsbergen et al. 1998), which seems to be mediated by altered signaling of vascular smooth muscle cells (Tuttle et al. 1995). Further studies also implicated increased expression of NGF in the vascular system as causing the development of sympathetic hyperinnervation resulting in hypertension (Emanueli et al. 2014). On the other hand, the activation of TrkA by NGF is also required for survival of the sympathetic and sensory neurons that innervate the heart (Emanueli et al. 2014). NGF can protect against post-ischemic dysfunction of sympathetic coronary innervation (Abe et al. 1997) and ischemia–reperfusion-induced myocardial injury in animal studies (Hiltunen et al. 2001). Therefore, it seems that in animal models NGF provides Janus-faced features mediating in one way the development of hypertension, and on the other hand, protecting against ischemia-induced injuries.

Recent human studies have reported that NGF has importance in atherosclerosis and related disorders (Chaldakov et al. 2004; Donovan et al. 1995). NGF level in advanced atherosclerosis of the coronary wall was significantly lower than in controls and plasma level of NGF was reduced in patients with metabolic syndrome compared with controls (Chaldakov et al. 2004). One of the possible ways for explaining the role of NGF in metabolic disorders is the fact that NGF upregulates the expression of LDL receptor-related protein, which leads to a decreased level of LDL, and subsequently a lower probability of atherosclerosis (Bu et al. 1998). Another explanation is that NGF shares a striking structural homology with proinsulin (Mukherjee and Mukherjee 1982), increases glucose-induced insulin secretion (Hiriart et al. 2001) and exerts certain insulin-like effects on lipid metabolism (Mukherjee and Mukherjee 1982; Ng and Wong 1985) and on energy homeostasis (Salton 2003). Taken together, the fact that plasma NGF level is reduced in chronic (Chaldakov et al. 2004) as well as in acute stages of human coronary atherosclerosis (Manni et al. 2005), suggests the role of NT deficiency in the pathophysiology of atherosclerosis and the association of NGF with CV diseases (Manni et al. 2005).

#### Direct psychosomatic evidences of NGF

No human study is available evaluating both psychopathological and CV disease conditions in relation with the role of NGF. The administration of NGF in eye-drops in streptozotocin-induced diabetic rats was proven to recover brain damage in the prefrontal cortex by activating protective and remodeling pathways triggered by BDNF. The authors also suggest based on their result that NGF-induced changes in BDNF signaling might influence the manifestation of depressive phenotype in diabetic rats (Rosso et al. 2017). These results suggest that the potentially beneficial psychosomatic effects of NGF are mediated by BDNF, but future studies are required to confirm this hypothesis.

#### Opportunities to restore NGF level

There are data in the literature that exercise can influence NGF. In soldiers, plasma NGF level was increased following emotional excitation and physical stress (Dugue et al. 1993; Schedlowski et al. 1993), and intense and prolonged physical exercise was associated with an increased serum NGF level in athletes (Bonini et al. 2013).

The therapeutic options for normalizing NGF levels are not that well known as in case of BDNF. Certain antidepressants—such as nortriptyline and citalopram—increased NGF levels in both experimental and clinical studies (Hassanzadeh and Rahimpour 2011). On the other hand, the decreased NGF level of patients with major depressive disorder decreased further after duloxetine administration (Martino et al. 2013). Interestingly, in another study after 8 weeks of intensive administration of antidepressants, significant improvement in the clinical symptoms of mood disorders was demonstrated without changes in NGF level (Liu et al. 2014). These results suggest neutral or bidirectional response of NGF for different antidepressant agents.

### Neurotrophin 3 (NT-3)

Neither NT-3 nor its proform, proNT-3 have received as much attention in the literature as BDNF or NGF and their proforms. NT-3 binds mainly to TrkC receptors, but can also interact with less efficiency with TrkA and TrkB receptors (Yano et al. 2009). Like other NTs, NT-3 has an important role in the development and maintenance of the nervous system. It stimulates and controls neurogenesis through the activation of TrkC receptors, plays role in the regulation of monoamine neurotransmitters such as noradrenaline and serotonin and enhances other neurotrophic factors such as BDNF and NGF (Pae et al. 2008). ProNT-3 exerts controversial effects inducing cell death in superior cervical ganglion neurons through binding a complex between sortilin and p75Ntr (Gibon and Barker 2017).

#### NT-3 in mood disorders

Studies about the possible connections between NT-3 and mood disorders are still controversial. Hock et al. found increased cerebrospinal fluid levels of NT-3 in elderly patients with depression compared with mentally healthy control subjects (Hock et al. 2000), and other studies also showed increased NT-3 level in bipolar disorders (Loch et al. 2015; Tseng et al. 2016). In contrast, a study demonstrated the opposite result (Otsuki et al. 2008), while Munkholm et al. found no difference in NT-3 level in bipolar disorder patients compared with controls (Munkholm et al. 2014).

#### NT-3 and CV pathology

NT-3 through TrkC activation is required for the development of the ventricles, the atria and the cardiac outflow tracts (Donovan et al. 1996; Tessarollo et al. 1997). The lack of NT-3 leads to septal defects and Fallot tetralogy (Donovan et al. 1996).

While BDNF through TrkB activation is required for the survival of arterial baroreceptors during development (Brady et al. 1999), NT-3 is also involved in the development of chemoafferent sensory neurons innervating the carotid body (Conover et al. 1995; Erickson et al. 1996).

It is known that the NT-3 receptor TrkC is expressed in mouse skeletal muscle endothelial cells and also in human veins. Cristofaro et al. found in animal models that NT-3 has a role in the stimulation of angiogenesis in which the endothelial NO synthase pathway has a critical role both in vivo and in vitro (Cristofaro et al. 2010).

Other studies suggest various effects of NT-3. Zhang et al. found elevated NT-3 level in hypertensive rat model compared with normotensive animals (Zhang and Rush 2001). The presence of NT-3 in human arteriosclerotic lesions was also observed (Donovan et al. 1995) and this NT seems to be a profibrogenic mediator in human aortic valve: overproduction of NT-3 by aortic valve tissue may contribute to the mechanism of valvular sclerosis (Yao et al. 2017).

#### Direct psychosomatic evidences of NT-3

No animal or human study is available evaluating the role of NT-3 in psychopathology and CV pathology at the same time. As common pathophysiological pathways are present similarly like in the case of other NTs, the clarification of the possible psychosomatic role of NT-3 could be an interesting task of future studies.

#### Opportunities to restore NT-3 levels

Similar to BDNF, NT-3 level can probably be restored with exercise: in animal model physical activity increased NT-3 and TrkC expression following focal cerebral ischemia (Chung et al. 2017).

Currently, available findings suggest possible therapeutic implications of NT-3 in the treatment of depression (Pae et al. 2008). This can be explained with the fact that NT-3 is a modulator of other NT-s, such as BDNF (Schutte et al. 2000) and has a direct effect on monoamine neurotransmitters such as 5-hydroxytryptamine and noradrenaline (Celada et al. 1996; Martin-Iverson et al. 1994).

### Neurotrophin 4 (NT-4)

Although NT-4 acts on the same TrkB receptor like BDNF, they do not invariably mediate the same effects (Hyman et al. 1994; Rodriguez Fermepin et al. 2009): BDNF elicits stronger and faster ubiquitination with TrkB than NT-4 (Proenca et al. 2016), and NT-4 has a more potent effect than BDNF in the protection on striatal dopaminergic neurons (Sauer et al. 1995). Little is known about proNT-4, but its truncated prodomain region prevents proNT-4 to bind to sortilin (Chen et al. 2005b). However, to date, its role in synaptic activity or synaptic plasticity has not been reported (Gibon and Barker 2017).

#### NT-4 in mood disorders

NT-4 promotes survival and differentiation of hippocampal (Ip et al. 1992, 1993), noradrenergic (Friedman et al. 1993) and dopaminergic (Altar et al. 1994; Hyman et al. 1994) neurons. Striatal dopaminergic neurons are believed to be one of the key neurons in the pathophysiology of bipolar disorder (Sauer et al. 1995), thus the association between mood disorders and NT-4 level is probable, but the results are controversial. Serum NT-4 was found to be elevated in patients with bipolar disorder compared with healthy controls (Tseng et al. 2016; Walz et al. 2009), however, in another study no differences were found between the two groups (Aydemir et al. 2014). Moreover, Barbosa et al. showed decreased NT-4 level in bipolar disorder with mania (Barbosa et al. 2014).

#### NT-4 and CV pathology

Similar to NT-3, NT-4 is also involved in the development of chemoafferent sensory neurons innervating the carotid body (Conover et al. 1995; Erickson et al. 1996), and its presence in human arteriosclerotic lesions was also shown (Donovan et al. 1995).

As TrkB is a mediator of the long-term blood pressure and sympathetic nerve activity responses to central angiotensin II activity (Becker et al. 2017), NT-4—binding the same TrkB-receptor—might have similar functions, even if the role of NT4/TrkB signaling in sympathetic control of blood pressure is still even less precisely understood than BDNF/TrkB signaling.

#### Direct psychosomatic evidences of NT-4

Similarly like NT-3, no animal or human studies are available evaluating the role of NT-4 in psychopathology and CV pathology at the same time. As NT-4 acts also on TrkB-receptor it might show similarities with the effects of BDNF, but the clarification of its exact role needs further studies.

#### Opportunities to restore NT-4 levels

Little is known about the effect of different medications on NT-4 level. In a study of Loch et al. in patients with bipolar disorder after the administration of lithium, NT-4 level did not change (Loch et al. 2015). Apart from these results, no data are available in the literature about the possibilities of NT-4 level modification and its physiological consequences.

## Conclusions

**Fig. 1:**
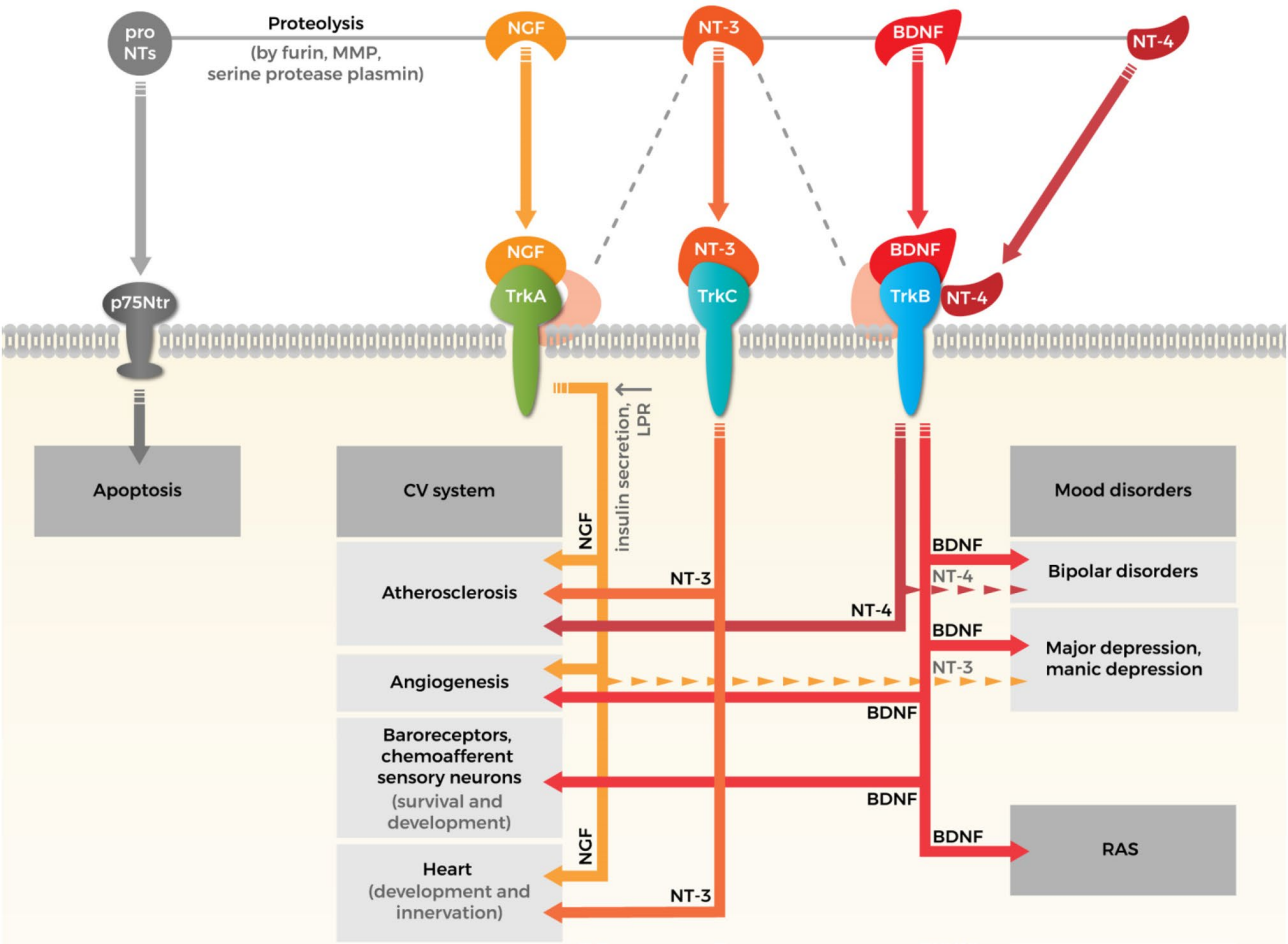
Crossroads of neurotrophins in cardiovascular system and psychopathology. Neurotrophin family consists four types of neurotrophins (NTs): nerve growth factor (NGF), brain-derived neurotrophic factor (BDNF), neurotrophin 3 (NT-3), and neurotrophin 4 (NT-4). NTs are synthesized as proforms that can be cleaved to release mature NTs. Both pro and mature forms of NTs are biologically active and eliciting opposite effects. ProNTs typically activate apoptotic downstream pathways via neurotrophin receptor p75 (p75Ntr). The effects of mature NTs are mediated by three tyrosine kinase receptors: NGF interacts with tropomyosin receptor kinase (Trk) A, BDNF binds to TrkB, NT-3 binds to TrkC and lower affinity to TrkA and TrkB (illustrated by gray dashed line) and NT-4 also interacts with TrkB. The effects of NTs on cardiovascular (CV) system: (i) NGF may have a protective role in atherosclerosis by upregulating LDL receptor-related protein (LPR) and increasing glucose-induced insulin secretion, while NT-4 and NT-3 seems to be a profibrotic mediator in aortic valve. (ii) Both BDNF and NGF promote angiogenesis through vascular endothelial cells directly or by influencing the action of other endogenous factors indirectly. (iii) BDNF is required for the survival of arterial baroreceptors. NT-3 is involved in the development of chemoafferent sensory neurons’ innervations of the carotid body. (iv) NGF promotes the survival of sympathetic and sensory neurons that innervate the heart. NT-3 promotes the development of the arteries and of the ventricles of the heart. The effects of NTs on mood disorders: (i) BDNF and NGF participate in the pathophysiology of depression: reduced levels of NGF and BDNF in serum and also in plasma have been demonstrated in patients suffering from depression. (ii) Association between mood disorders and NT-3, NT-4 is plausible, but results are still controversial (illustrated by dashed narrows). Renin–angiotensin system (RAS) is one of the possible pathways might explaining the association between BDNF and CV function and susceptibility to mental disorders

The accumulating data about the role of NTs, especially BDNF both in psychopathology and CV diseases, suggest that besides the involvement of the hypothalamic–pituitary–adrenal axis and the autonomic nervous system, NT pathway can also mediate psychosomatic processes. Its physiological background is based on the shared signaling pathways descending from Trk receptors and p75Ntr to both psychopathological and CV directions. The summary of our knowledge in this field, which was detailed above in this manuscript is demonstrated in Fig. 1.

However, much more studies are needed, not only observational, but also interventional ones evaluating in psychopathological and CV conditions at the same time aiming to discover the mediator role of NTs to confirm this hypothesis, but in case of supportive results, new therapeutic targets can be defined with the possibility of beneficial effects on two groups of diseases with huge public health impact.
